# Relevance of polymorphisms in *MC4R* and *BDNF* in short normal stature

**DOI:** 10.1186/s12887-018-1245-1

**Published:** 2018-08-22

**Authors:** Nikolas Herrfurth, Anna-Lena Volckmar, Triinu Peters, Gunnar Kleinau, Anne Müller, Cigdem Cetindag, Laura Schonnop, Manuel Föcker, Astrid Dempfle, Stefan A. Wudy, Struan F. A. Grant, Thomas Reinehr, Diana L. Cousminer, Johannes Hebebrand, Heike Biebermann, Anke Hinney

**Affiliations:** 1 Department of Child and Adolescent Psychiatry, Psychosomatics and Psychotherapy, University Hospital Essen, University of Duisburg-Essen, Essen, Germany; 2 Institute of Experimental Pediatric Endocrinology, Charité – Universitätsmedizin Berlin, corporate member of Freie Universität Berlin, Humboldt-Universität zu Berlin, and Berlin Institute of Health, Berlin, Germany; 3 Present Address: Group Protein X-ray Crystallography and Signal Transduction, Institute of Medical Physics and Biophysics, Charité – Universitätsmedizin Berlin, corporate member of Freie Universität Berlin, Humboldt-Universität zu Berlin, and Berlin Institute of Health, Berlin, Germany; 4 Institute of Medical Informatics and Statistics, Christian-Albrechts University Kiel, Kiel, Germany; 5 Division of Pediatric Endocrinology and Diabetology, Center of Child and Adolescent Medicine, Giessen, Germany; 6 Divisions of Human Genetics and Endocrinology, Children’s Hospital of Philadelphia Research Institute, Philadelphia, USA; 7 Department of Pediatric Endocrinology, Diabetes and Nutrition Medicine, Vestische Hospital for Children and Adolescents Datteln, University of Witten/Herdecke, Datteln, Germany; 8 Department of Genetics, University of Pennsylvania, Philadelphia, USA

**Keywords:** Function, Hypothalamus, Weight regulation

## Abstract

**Background:**

Variation in genes of the leptinergic-melanocortinergic system influence both body weight and height. Because short normal stature (SNS) is characterized by reduced body height, delayed maturation and leanness, allelic variation of genes in this pathway are hypothesized to affect this common condition.

**Methods:**

We analyzed the coding regions of *LEP*, *MC4R*, *MRAP2* and *BDNF* in 185 children with SNS (height < 5th percentile) to search for non-synonymous and frameshift variants. For association studies (two-sided χ^2^-tests) population-based data sets (ExAC, EVS and KORA) were used. Cyclic AMP accumulation, cell surface expression, central expression and MAP kinase activation were assayed in vitro to determine the functional implications of identified variants.

**Results:**

We detected eleven variants predicted to be protein-altering, four in *MC4R*, four in *BDNF*, and three in *MRAP2*. No variants were found in *LEP*. In vitro analysis implied reduced function for the MC4R variant p.Met215Ile. Loss-of-function is contrary to expectations based on obesity studies, and thus does not support that this variant is relevant for SNS. The minor SNP alleles at MC4R p.Val103Ile and BDNF p.Val66Met were nominally associated with SNS.

**Conclusion:**

Taken together, although genes of the leptinergic-melanocortinergic system are important for normal growth, our data do not support the involvement of rare mutations in *LEP*, *MC4R, MRAP2* or *BDNF* in short normal stature.

**Electronic supplementary material:**

The online version of this article (10.1186/s12887-018-1245-1) contains supplementary material, which is available to authorized users.

## Background

Short stature is one of the most common reasons for referral of children to pediatric endocrinology departments. In up to 80% of cases there is no identifiable cause for the phenotype [1]. Short normal stature (SNS) is defined as a body height below the age- and sex- specific 5th percentile and by the absence of readily detectable pathogenic causes like illness, hormonal deficiency or dysmorphic syndromes [2].

Human height is a complex, highly heritable and polygenic trait. A large genome-wide association study (GWAS) meta-analysis identified 697 genome-wide significant variants at 423 different loci by analysis of a population-based sample of 253,288 individuals. Together these variants explain about 20% of the heritability of adult height variation [3]. Recently a GWAS focussed on the relevance of rare and low-frequency variants on human adult height variation. Some variants with lower minor-allele frequencies revealed effects of up to 2 cm per effect allele. Together all variants explained 27.4% of the heritability of body height [4].

The leptinergic-melanocortinergic-pathway, which includes leptin (*LEP*), the melanocortin 4 receptor (*MC4R*), melanocortin receptor accessory protein 2 (*MRAP2*) and brain derived neurotrophic factor (*BDNF*)*,* is involved in the regulation of both body height [3, 5–9] and weight [6, 10]. Loss-of-function mutations affecting of the components of this system lead to obese phenotypes with increased linear growth [6, 8, 9].

The function of each of these genes provides evidence that they may be candidates for playing a causal role in SNS. (1) Rare homozygous loss-of-function mutations in the *LEP* gene [10] are associated with hypogonadism, frequent infections and severe early onset obesity [11, 12]. A rare non-synonymous mutation located in a highly conserved *LEP* position was detected in a boy with short stature and his mother. Both heterozygous carriers shared a similar phenotype of reduced appetite, pubertal delay and leanness [13]. (2) Up to now more than 160 non-synonymous, nonsense or frameshift mutations in *MC4R* resulting in reduced receptor function have been described. Carriers of these mutations are mostly (extremely) obese, hyperphagic, hyperinsulinemic and display increased linear growth [5, 6, 14]. The minor alleles at two *MC4R* polymorphisms (rs2229616 [p.Val103Ile] and rs52820871 [p.Ile251Leu]) are associated with slightly reduced body weight [15, 16]. rs17782313, identified in GWAS of BMI/obesity [17] and located 3′ to *MC4R,* is also associated with human adult height (*p* = 3.80 × 10^− 11^) [7]. Mc4r-deficient mice are obese with increased length [18], in cavefish Mc4r mutations lead to binge eating and increased body length [19]. An artificially induced increase in MC4R activity in the early development of zebrafish embryos causes growth retardation [20]. (3) MRAP2 influences MC4R signalling. A mutation screen in obese children and adolescents revealed that *MRAP2* variants might contribute to human obesity [8]. Additionally, we described an *MRAP2* mutation leading to reduced MC4R function [21]. Animal models demonstrate the impact of Mrap2 on metabolism, growth and development [22]. Mice with germline deletion of Mrap2 are characterized by obesity and increased linear growth [22]. (4) BDNF regulates, mediated by the TrkB receptor, energy homeostasis downstream of MC4R [23]. In humans, the association of a BDNF variant was described for childhood BMI, weight and height standard deviation score (SDS) [24]. Conditional brain-specific *Bdnf* knockdown resulted in increased body weight and linear growth [9]. TrkB hypomorphic mice also showed a phenotype characterized by obesity and increased linear growth [23].

The phenotype comprising reduced linear growth and leanness in children with SNS [25] led us to hypothesize that gain-of-function variants in *LEP*, *MC4R*, *MRAP2* and/or *BDNF* might influence this condition. Hence, we screened the coding regions of the respective genes for mutations in 185 children with SNS.

## Methods

### Study groups

We screened 185 (120 male) unrelated children (age 12.08 ± 3.61 years, height percentile 1.60 ± 1.33, BMI 17.56 ± 2.83 kg/m2, BMI percentile 36.52 ± 26.41) with SNS. SNS was defined as height below the 5th percentile for age and sex which is not due to illness, hormonal deficiency or part of a dysmorphic syndrome. To distinguish SNS from other types of short stature, children with dysmorphic features or chronic diseases were excluded. The following laboratory parameters were measured to rule out chronic inflammation (erythrocyte sedimentation rate, blood count, C-reactive protein), celiac disease (gliadin and endomysium antibodies), hepatic disease (aspartate aminotransferase, alanine aminotransferase), or renal disease (creatinine) and hypothyroidism (free thyroxin, thyrotropin). Growth-hormone deficiency was considered to be unlikely based on insulin-like growth factor–binding protein 3 (IGFBP-3) levels and serum insulin-like growth factor I (IGF-I) [25, 26]. Probands with SNS were recruited together with their biological parents and affected siblings if available between November 2001 and March 2007 at the endocrine outpatient unit of the Children’s hospital in Gießen (Germany) [25, 26]. Growth velocity in the short stature children was normal according to routine assessement at pediatricians and measurements at up to three different time points (admission, 1st and 2nd referral).

For association studies of detected exonic SNPs with SNS, population-based data sets were used. The Exome Aggregation Consortium (ExAC) comprises exome data of 60,706 unrelated individuals sequenced as part of various genetic studies (http://exac.broadinstitute.org/, accessed on June 2nd 2015). The Exome Variant Server (EVS) is based on the NHLBI GO Exome sequencing project (ESP). We used genotype data from a total of 4300 participants from the European American population (http://evs.gs.washington.edu/EVS/accessed on June 2nd 2015). Additionally, genotype data from 7937 participants of two population based surveys from KORA (Cooperative Health Research in the Region Augsburg; [27]) was used. The intronic SNP rs17782313 was compared with data from the dbSNP database (http://www.ncbi.nlm.nih.gov/SNP/index.html).

### Mutation screening

Sanger re-sequencing, denaturing high performance liquid chromatography (WAVE, [28]) and high resolution melting (HRM, [29]) were used to detect variations in the coding regions of *LEP, MC4R, MRAP2* and *BDNF* (for details see Additional file 1: Table S1)*.*

### Conservation

Conservation was analyzed by aligning the sequences of 55 (*MC4R*) and 61 (*BDNF*) species in total using orthologous data from the database Ensembl (http://www.ensembl.org/index.html, accessed on April 26th 2015, see Additional file 1: Figure S1).

### In vitro analyses

The rare novel MC4R variant p.Met215Ile was analysed in vitro (cAMP accumulation assay; MAP kinase activation via serum response element (SRE) luciferase reporter gene assay and cell surface expression; for details see Additional file 1: Text S1).

### Expression analyses

**Fig. 1:**
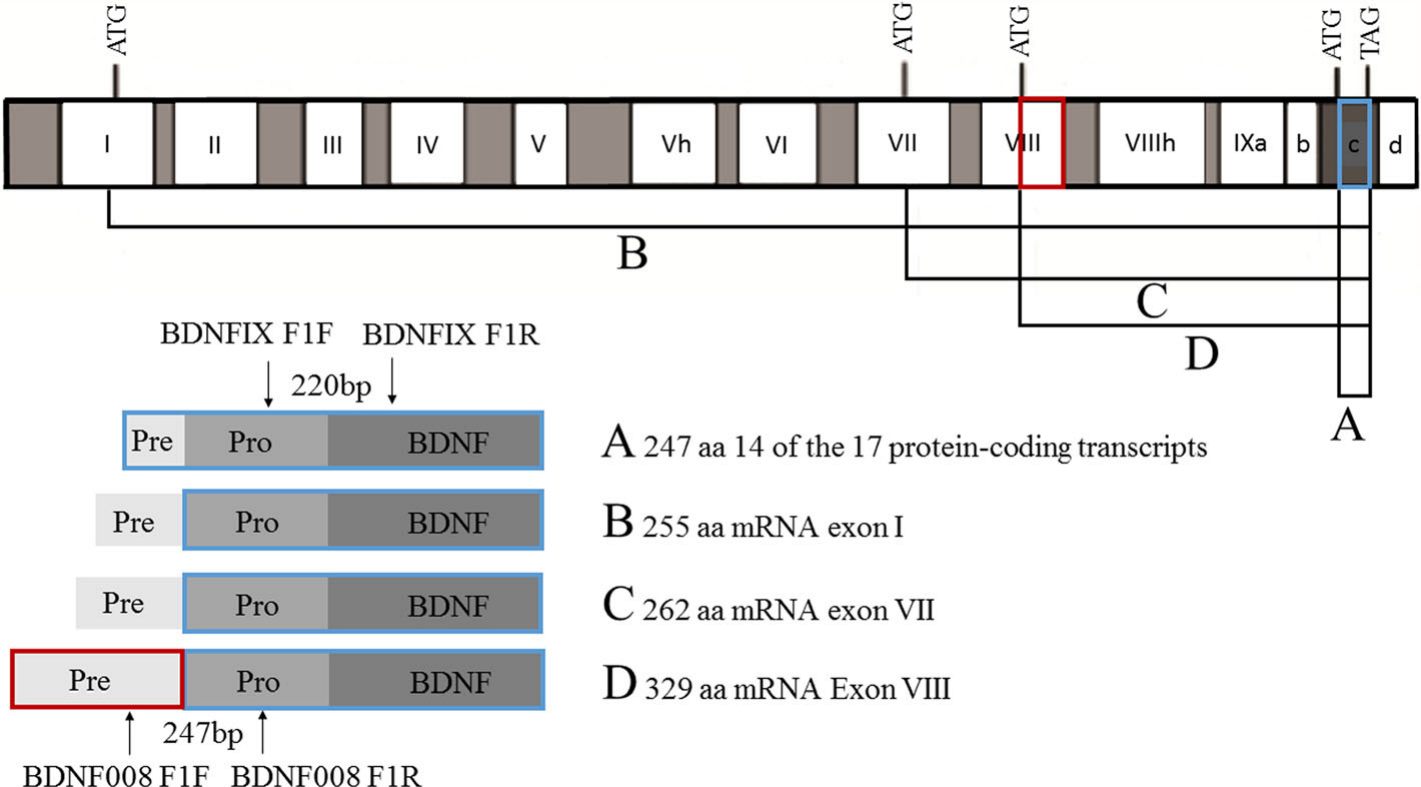
Analysed BDNF transcripts. Schematic representation of the *BDNF* transcripts and primer positions for the *BDNF* expression analysis. Blue boxes mark the analysed *BDNF* exon IX, and a red box marks the analysed *BDNF* exon VIII (modified from [53])

The amount of total *BDNF* mRNA (proBDNF) and the longest mRNA (pre-pro-BDNF isoform, Chr. 11: 27,654,893-27,701,053; ENST00000438929; Ensembl) in the human brain were analyzed with a human brain cDNA array (Tissue Scan Human Brain Tissue qPCR Array HBRT101, Origene, Rockville, MD, USA). To amplify specifically the longest BDNF transcript, primers were designed to include the exon-intron boundaries of *BDNF* exons VIII and IX (see Fig. 1). The amount of the core *BDNF* transcripts (including all splice forms) was analyzed by a PCR fragment within *BDNF* exon IX. *GAPDH* served as a housekeeping control gene [30] (for details see Additional file 1: Table S2). Expression analysis studies were performed on the Tissue Scan Human Brain Tissue qPCR Array HBRT101 (Origene, Rockville, MD, USA) comprising 24 different brain tissues. qPCR was conducted with RT^2^ SYBR® Green Rox™ qPCR Mastermix (Qiagen) according to the manufacturer’s instructions.

### Statistical analyses

For association analysis, we used published data from epidemiological studies (see above). To test for association between identified variants and SNS, two-sided χ2-tests were used and nominal *p*-values are given. Correction for multiple testing was not performed. Hardy-Weinberg equilibrium was tested in the SNS study group. For cAMP accumulation and MAP kinase activation, data analysis and statistics were done using GraphPad Prism (GraphPad Software, San Diego, CA, USA). An uncorrected p-value of 0.05 was used to denote nominal significance. The statistical difference in maximal stimulation analyses was calculated by a t-test with Welsh correction.

## Results

Mutation screens of the coding regions of *LEP*, *MC4R*, *MRAP2* and *BDNF* revealed a total of 11 variants (Table 1). We did not further analyse *MRAP2,* as only synonymous variants were detected, and *LEP,* as we did not detect variation in the coding region.

**Table 1:** Detected non-synonymous and frameshift variants in the protein coding regions of *LEP, MC4R*, *MRAP2* and *BDNF* in 185 index patients with short normal stature

Gene	rs-number	aa-exchange	variant carrier (%)/MAF (%) SNS	MAF (%) EVS	MAF (%) ExAC All^a^/European^b^	Conservation
LEP	None	None	–	–	–	–
MC4R	None	p.Met215Ile	1 (0.54%) /0.27	None	0.0008/0.0014	98.2%
	rs13447329	p.Thr112Met	2 (1.08%) /0.54	0.07	0.12/0.20	70.9%
	rs52820871	p.Ile251Leu	4 (2.16%) /1.08	1.16	0.68/1.00	78.2%
	rs2229616	p.Val103Ile	12 (6.49%) /3.24	1.85	1.74/1.95	94.5%
BDNF	rs8192466	p.Thr2Ile	1 (0.54%) /0.27	0.40	0.11/0.25	93.4%
	rs6265	p.Val66Met	79 (42.70%) /23.51	19.07	19.37/19.29	93.4%
	rs539177035	p.Cys34PhefsTer12	1 (0.54%) /0.27	None	0.0054/0.0050	n.a.
	rs551669106	p.Val56Ala	1 (0.54%) /0.27	None	0.01/0.14	8.2%
MRAP2	None	None	–	–	–	–

*aa* amino acid, *MAF* minor allele frequency, *SNS* short normal stature, *EVS* Exome variant server based on the Exome Sequencing project NHCBI (http://evs.gs.washington.edu/EVS/) for approximatly 4300 European Americans; ExAC (http://exac.broadinstitute.org/about) comprising ^a^60,706 unrelated individuals from different ethnic background; of these approximatly ^b^34,000 are of European (without Finland) descent; conservation analysis was performed by aligning sequences of 55 (MC4R) and 61 (BDNF) species in total using orthologous data from the database Ensembl (http://www.ensembl.org/index.html; September 23th 2016)

### MC4R

**Fig. 2:**
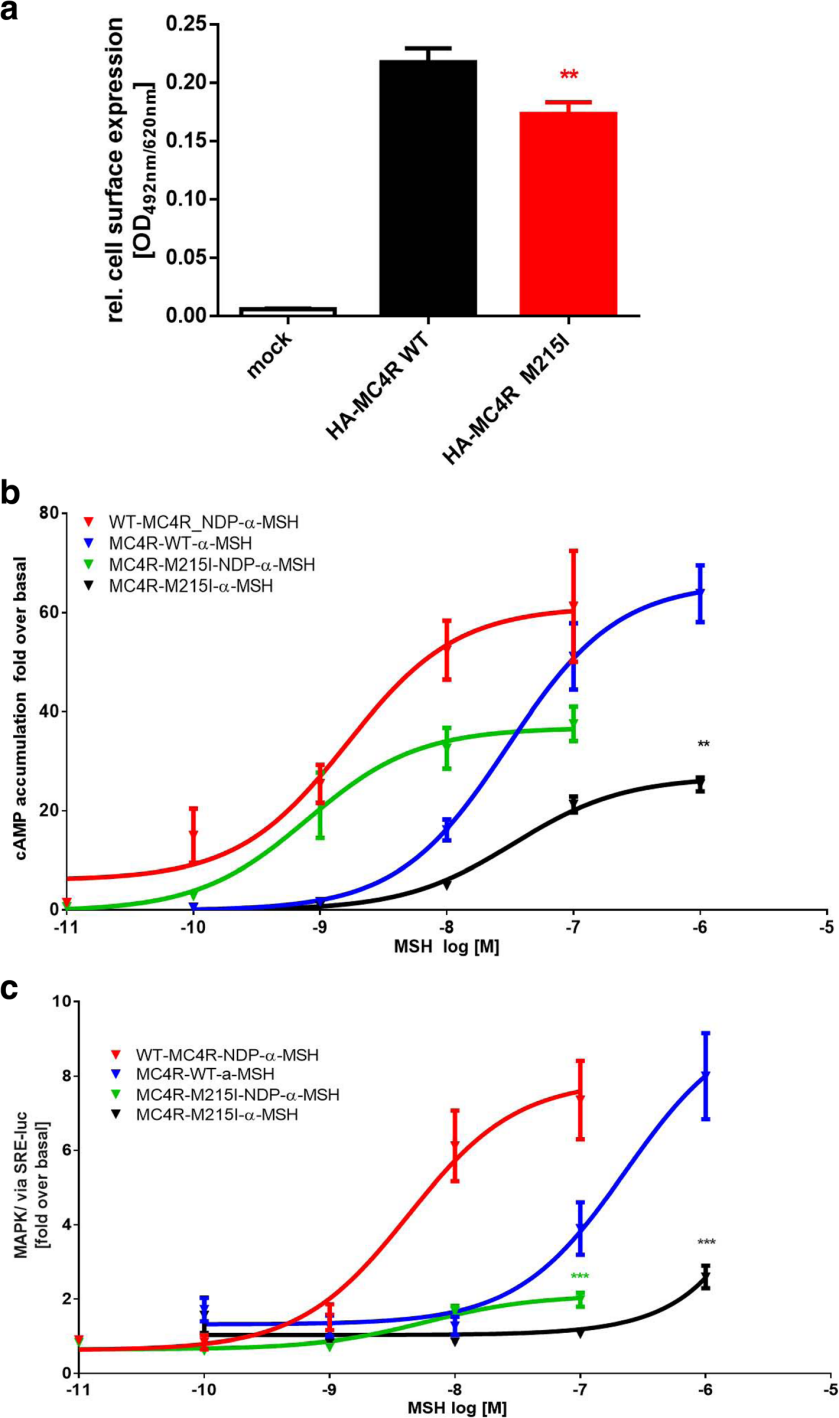
Results of in vitro analyses of MC4R variant p.Met215Ile - **a**) cell surface expression, **b**) cAMP accumulation, C) MAPK/ERK assay HEK293 cells for cAMP accumulation and MAP kinase determination (**b**, **c**) and COS-7 cells for cell surface ELISA (**a**) were transfected as indicated in the Methods section. **a** Cell surface ELISA with N-terminally HA tagged receptors show a slight reduction in cell surface expression compared to the wild-type. The result of five independent experiments performed in sextuplicate is shown. Data represents mean ± SEM. A test with Welsh correction was performed for statistical analysis comparing wild-type to p.215I. **b** cAMP accumulation after stimulation with increasing amounts of NDP-α-MSH and α-MSH stimulation indicate a loss of maximal stimulation of p.M215I. EC50 values for alpha-MSH induced signaling for wt-MC4R and MC4R-M215I are 30 nM and 33 nM, respectively and for NDP-α-MSH induced signaling 1.6 nM and 0.7 nM. The result of four independent experiments performed in triplicate is shown. Data were calculated as fold level over basal stimulation and are indicated as mean ± SEM. The statistical difference in maximal stimulation was calculated by a t-test with Welsh correction. **c**: MAP kinase signalling was determined using an SRE-luciferase reporter gene assay after stimulation with increasing amounts of NDP-α-MSH and α-MSH stimulation. M215 resulted in a complete loss-of-function for stimulation with NDP-α-MSH and α-MSH α-MSH so that calculation of EC50 values is impossible. EC50 value for wt-MC4R after α-MSH or NDP-α-MSH challenge are 229 nM and 4.2 nM, respectively. The result of four independent experiments performed in triplicate is shown. Data were calculated as fold level over basal stimulation and are indicated as mean ± SEM. The statistical difference in maximal stimulation was calculated by a t-test with Welsh correction

**Fig. 3:**
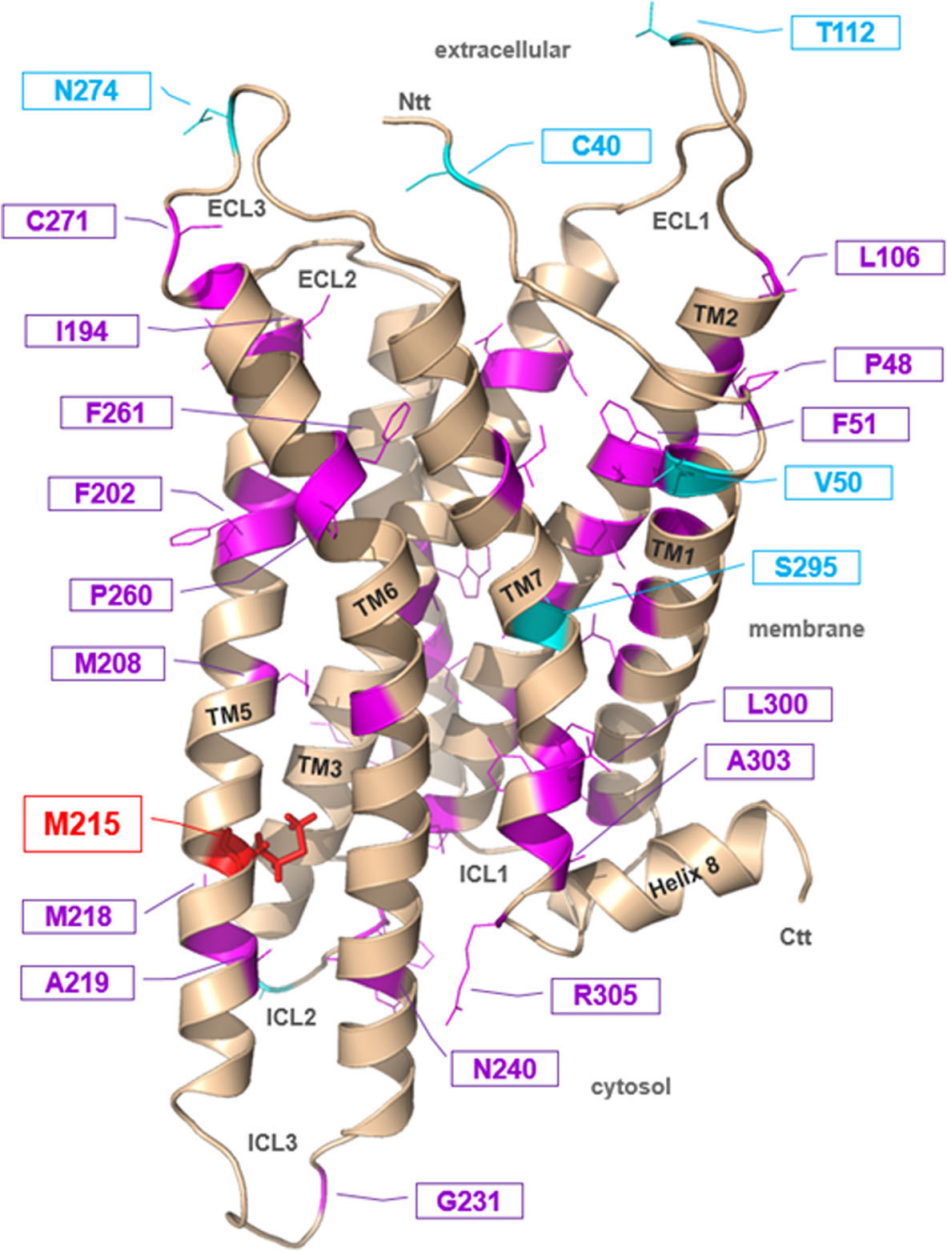
Structural homology model of MC4R with positions of naturally occurring mutations. MC4R homology model [58] was used to visualize the wild-type positions of known pathogenic single side-chain substitutions (according to the review of [31], magenta side-chains, only few are labeled as examples). The MC4R mutant p.Met215Ile is highlighted. The methionine (red stick side-chain) is located in the transmembrane (TM) helix 5. Several mutations are reported to have selective influence on different signalling pathways induced by MC4R [31]. Those which are characterized by a selective impairment of the ERK pathway are coloured in cyan

Four non-synonymous variants in *MC4R* were identified (Table 1). The rare non-synonymous, conservative *MC4R* variant p.Met215Ile was heterozygous in an 11.13-year-old male. The substitution is at a highly conserved position (98.2%; 54 out of 55 species show the human wild type amino acid at this position, Additional file 1: Figure S1). In vitro studies revealed differences between wild-type and p.215Ile MC4R (Fig. 2a). Cell surface expression was slightly but significantly reduced to 80% of wild-type expression for p.215Ile MC4R. cAMP accumulation was nominally reduced after NDP-α-MSH challenge for p.215Ile. However, EC50 values of the mutation were slightly improved (1.6 nM for wild-type MC4R and 0.7 nM for p.215Ile, Fig. 2b). Stimulation with the endogenous ligand α-MSH revealed a strong reduction of maximal signalling to 39.6% of wild-type MC4R signalling, but nearly identical EC50 values (30 nM for wild-type and 33 nM for p.215Ile) were observed (Fig. 2b). Investigation of MAP kinase signalling indicated a complete loss-of-function for p.215Ile, which did not allow proper calculation of EC50 values for the mutation. Wild-type MC4R signalling resulted in an EC50 of 4.2 nM for NDP-α-MSH and 229 nM for α-MSH (Fig. 2c). This finding indicates a much stronger effect of the mutation on MAP kinase signalling compared to Gs/adenylyl cyclase activation. The amino acid methionine at position 215 is highly conserved and is located at a hot spot for receptor function related to the activation mechanism (Fig. 3). The slight trend toward improved signaling in Gs/adenylyl cyclase activation and the complete loss of function in terms of MAPK activation of the MC4R-215Ile mutation indicates differential signaling capacities (also known as biased signaling [31]) of the mutant. From these in vitro data we could only speculate that the net effect of both signaling pathways of this variant results in loss of function.

The *MC4R* variant rs13447329 (p.Thr112Met) was heterozygous in two unrelated probands (Additional file 1: Table S4). This variant leads to a non-synonymous, non-conservative substitution at a conserved position (70.9%, Additional file 1: Figure S1). The 15-year-old male adolescent had a delayed bone age (bone age retardation: − 2.28 years), while the 17-year-old female adolescent had a reduced leptin level of 6.4 μg/L (leptin SDS: − 0.90). Her bone age was also slightly delayed (bone age retardation: − 0.27 years). Most functional in vitro analyses showed a similar function as the wild type MC4R, while some implicated a reduced function [14]. As the variant was frequently detected among obese and normal weight and height individuals [14], a role in SNS is unlikely.

Two *MC4R* polymorphisms rs52820871 (p.Ile251Leu) and rs2229616 (p.Val103Ile) were also detected. Four children were heterozygous for the non-synonymous polymorphism p.Ile251Leu. This SNP leads to a conservative amino acid substitution and is located at a conserved position (78.2%, Additional file 1: Figure S1). The non-synonymous, conservative polymorphism rs2229616 (p.Val103Ile) was heterozygous in a total of 12 subjects. This polymorphism is located at a highly conserved position (94.5%, Additional file 1: Figure S1). For both minor alleles (251Leu and 103Ile, respectively), a slightly increased MC4R function has been described [14–16, 32], which is concordant with a weight-lowering effect.

For association analyses, we used published data from large-scale population-based sequencing projects as controls (Additional file 1: Table S3). We assumed that body height is normally distributed within these cohorts [27]. For p.Ile251Leu, we detected no association (p.Ile251Leu variant carrier frequency in SNS: 2.16%, ExAC 2.00%, nominal *P* = 0.87; EVS 2.33%, nominal *P* = 0.89, Additional file 1: Table S3), but for p.Val103Ile, the variant carrier frequency in SNS (6.49%) was nominally higher compared with data from KORA (3.67%; nominal *P* = 0.045), EVS (3.70%; nominal *P* = 0.052) and ExAC (3.86%; nominal *P* = 0.065). The largest published meta-analyses for human adult height did not show an association for the infrequent allele at p.Val103Ile ([3, 4]). In children and adolescents, no supporting evidence from flanking markers with height could be observed among 4556 subjects [33]. Genotyping of the GWAS-derived SNP (*P* = 3.8 × 10^− 11^) for human adult body height (rs17782313) near the *MC4R* gene in our 185 SNS probands (MAF = 0.25) did not confirm the association to body height (dbSNP: Hapmap CEU European MAF = 0.265 *P* = 0.68).

### BDNF

In *BDNF* we detected four non-synonymous variants. Variation rs8192466 (p.Thr2Ile) leads to a non-conservative, non-synonymous substitution at a highly conserved position (93.4%, Additional file 1: Figure S1). The variant was heterozygous in an 11-year-old boy with SNS (Additional file 1: Table S4). The affected child had a slight bone age retardation (− 0.57 years) and an increased leptin level of 5.02 μg/L (leptin SDS: 2.03). The boy inherited the p.2Ile variant from his mother. His 4-year-old brother carried the p.2Ile variant as well, whereas the father was a non-carrier. Additionally, all four analysed family members were heterozygous for the *BDNF* polymorphism rs6265 (p.Val66Met).

The conservative, non-synonymous polymorphism rs6265 (p.Val66Met) was detected in a total of 79 children with SNS (71 heterozygous; 8 homozygous). The amino acid position 66 is highly conserved (93.4%; Additional file 1: Table S1). 66Met is nominally associated with SNS (MAF = 23.51%) in comparison with ExAC (MAF = 19.26%; nominal *P* = 0.040) and EVS (MAF = 19.07%; nominal *P* = 0.034, Additional file 1: Table S3). Again, both comparisons showed a directionally consistent effect. A large meta-analysis for human height variation just failed to show nominal association for the infrequent allele at p.Val66Met with human adult height (*P* = 0.063; [3]). However, the direction of the effect is the same as above. In children with childhood obesity [33], p.Val66Met was also nominally associated with height (*P* = 0.045).

**Fig. 4:**
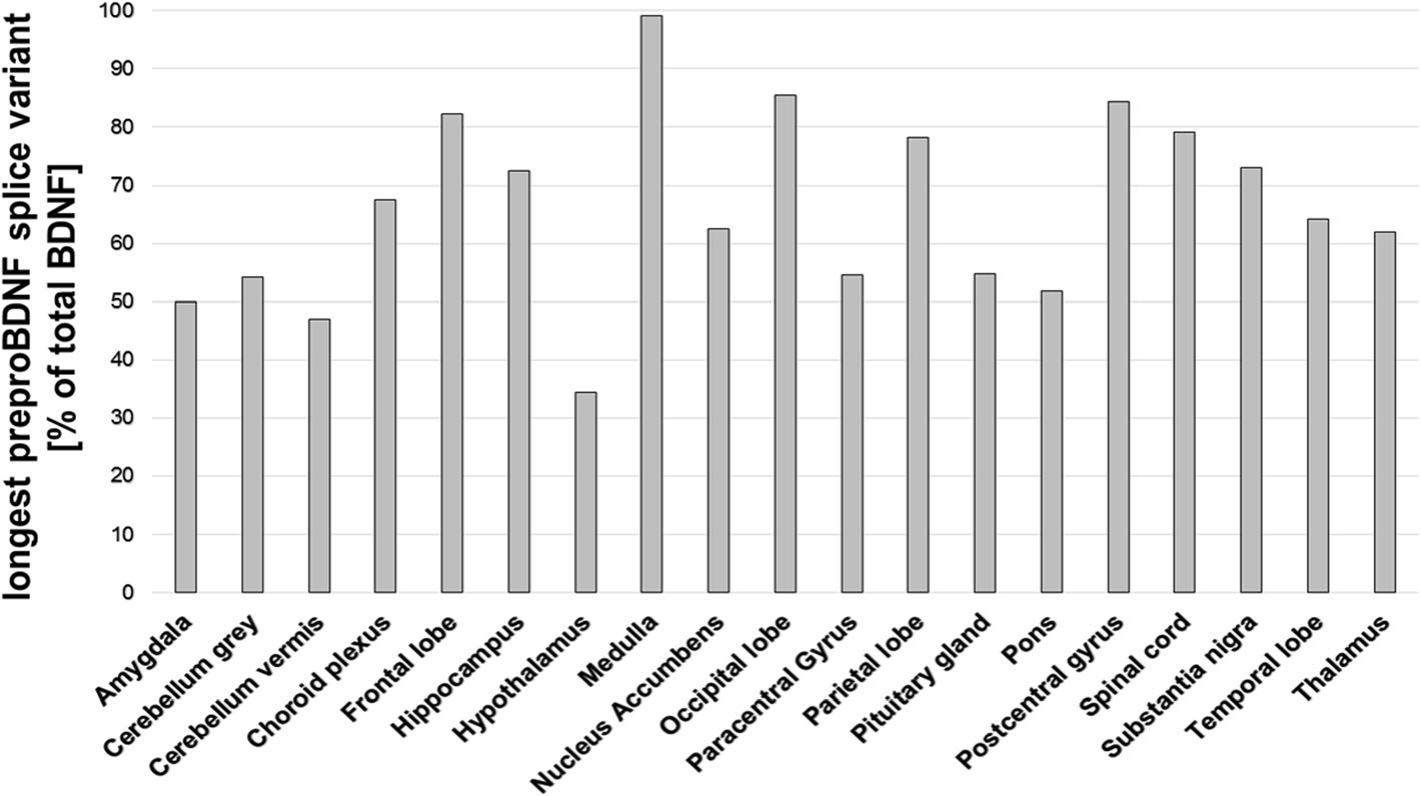
*BDNF* transcript expression analysis. To analyze the amount of total *BDNF* mRNA (proBDNF) and the longest mRNA (pre-pro-BDNF isoform, Chr. 11: 27,654,893-27,701,053; ENST00000438929; Ensembl) in the human brain, a cDNA array (Tissue Scan Human Brain Tissue qPCR Array HBRT101, Origene, Rockville, MD USA) was used which comprises 24 different brain tissues, 19 of which were positive for *BDNF* transcripts (the long and core versions)

Additionally, we found two rare mutations, rs539177035 (p.Cys34PhefsTer12) and rs551669106 (p.Val56Ala) in the longest splice form of *BDNF* (pre-pro-BDNF isoform, BDNF-008, ENST00000438929, Ensembl). As this splice form was not well characterized, we screened a brain-derived cDNA library to analyse the central expression pattern for the splice form. *BDNF* was ubiquitously centrally expressed (19 of 24 different brain tissues were positive). For some regions (e.g. the medulla), BDNF-008 is the main transcript (Fig. 4). In the hypothalamus, the longest pre-pro-BDNF represented about one third of total *BDNF* transcripts.

We detected an infrequent frameshift mutation, rs539177035 (p.Cys34PhefsTer12), in a 10.3-year-old boy with normal Tanner stage [34]. He had a retarded bone age (− 3 years) and a normal leptin level (1.74 μg/L; leptin SDS: − 0.21). Both his father and 7-year-old brother were heterozygous for this variant, while his mother was a non-carrier. This frameshift mutation leads to an altered reading frame from amino acid position 33. The mutated protein terminates after amino acid 44.

The conservative previously unknown non-synonymous mutation rs551669106 (p.Val56Ala) was heterozygous in a child with SNS (see Table 1). The boy inherited the mutation from his mother. Phenotype data from his mother was not available. The amino acid position 56 shows low conservation (8.2%, Additional file 1: Figure S1).

## Discussion

There is evidence for an involvement of the leptinergic-melanocortinergic system in body height and weight regulation. Hence, we screened the coding regions of major players in this system (*LEP, MC4R*, *MRAP2* and *BDNF*) for mutations in a total of 185 unrelated children with SNS.

### MC4R - relevance of p.Met215Ile and polymorphisms in short normal stature

The rare *MC4R* mutation p.Met215Ile was identified in addition to three previously known variants. This non-synonymous variant was detected in a lean boy with SNS. Methionine at this position is highly conserved and in relation to the activation mechanism it is located at a hot spot for receptor function (Fig. 3). In detail: Several mutations have already been reported to (selectively) influence signalling pathways transmitted by MC4R [31] and especially in transmembrane domain 5 (TM5) many pathogenic mutants are already known. Mutation p.Met215Ile is in close proximity e.g. to p.Met208 and p.Met218, where pathogenic mutants were also reported (Fig. 3). Interestingly, the 3-dimensional perspective on positions of pathogenic variants revealed that the known substitutions are distributed over the entire receptor and at each transmembrane helix. Hot-spots are TM2, 4, and 5. p.Met215 is not located at the direct ligand binding site close to the extracellular region and between the extracellular loops (ECLs). This implies that the mutant p.Met215Ile does not disturb the process of ligand-binding. The model rather suggests that p.Met215 is at a critical region of helix arrangements (interfaces between TM3/5/6) and is - in the inactive state conformation - tightly embedded in a hydrophobic cage constituted by amino acids from TM3 and TM6. This region is modified during the process of receptor activation, which is a prerequisite to (fully) bind G-protein or arrestin [35, 36]. It can be assumed, therefore, that any change at this position concerning side-chain volume and biochemical properties will consequently lead to restrictions in the signalling capacity. This is also reflected by the high conservation of this residue among compared sub-species (Additional file 1: Figure S1).

*MC4R* mutations leading to a reduced function are mainly found in (extremely) obese individuals [14]. As linear growth in obese adolescents is increased [6], we hypothesized that the p.215Ile variant leads to a gain of MC4R function in a lean and small child. However, our in vitro results and in silico analyses (Fig. 4) both implied a reduced MC4R function.

Additionally, we identified three non-synonymous variants in the *MC4R* gene (p.Thr112Met, p.Ile251Leu and p.Val103Ile), which were previously described in both normal weight/height and obese individuals [6, 14, 37, 38]. Two of these polymorphisms (p.Ile251Leu and p.Val103Ile) protect from obesity [15, 16], which results from improved receptor function [39]. We found nominal association of the infrequent MC4R 103Ile allele with SNS. A lookup of this variant in the largest GWAS meta-analysis for adult height (*n* = 253,288 [3]), however, did not support this association. In a GWAS for body height in children and adolescents, an association with height was not detected [33]. We also did not find association of rs17782313, located 188 kb downstream of *MC4R,* in our children with SNS, although association with human height has been reported in GWAS [7, 17]. Interestingly, SNP rs5030980 in the second intron of *AgRP* reached genome wide significance in the recent GWAS for human body height variation [4] AgRP is an endogenous, inverse agonist at the MC4R. AgRP-variants could lead to an attenuated inhibition resulting in increased MC4R-function [14]. These findings underscore the relevance of the melanocortinergic system for body height.

### BDNF variants in short normal stature

The *BDNF* screen revealed four non-synonymous variants. A boy with SNS carried the previously detected non-conservative variant p.Thr2Ile. Additionally, his small-normal weight mother and brother also carried this mutation. This amino acid position is conserved across different species (93.4%, see Additional file 1: Figure S1). p.Thr2Ile was previously identified in a boy with idiopathic congenital central hypoventilation syndrome (CCHS). His heterozygous father showed symptoms of autonomic nervous system dysfunction but not of CCHS [40]. Both syndromes are severe, are accompanied by gastro-oesophageal dysfunctions and can lead to reduced growth and development [41]. However, in our SNS probands, postnatal abnormalities were not reported. Additionally, association of the p.2Ile allele with weight regulation has not yet been detected [42, 43]. The frequency of the p.2Ile allele in our SNS study group (0.54%) is comparable with other studies (0.55%; [42]; 0.53%; [43]), hence a relevance in weight regulation and SNS is unlikely.

The infrequent allele at p.Val66Met is nominally associated with SNS. A GWAMA (GWAS meta analysis) for adult height in population-based individuals [3] found a directionally consistent, but non-significant, trend (*P* = 0.063) for this allele with body height. In the Early Growth Genetics childhood obesity dataset, p.Val66Met reached nominal significance (*P* = 0.045) and was also directionally consistent. This polymorphism was previously described and is related to several clinical traits including obsessive-compulsive disorders [44], bipolar affective disorders [45], Parkinson disease [46] and eating disorders [47, 48], in some but not all studies [42, 49, 50].

p.Val66Met is expressed in the pro-domain of BDNF. In vitro studies showed a functional relevance for the minor allele [51]. The 66Met allele affects the secretion and intracellular processing of BDNF by the activity dependent pathway and affects hippocampal function [52]. Only little is known about the biological function of proBDNF, although recently it was shown that it facilitates hippocampal long term depression (LTD). p.Val66Met completely inhibits LTD in the hypothalamus [51]. There are three final paths for pro-BDNF: it can (i) be modified in the Golgi and be secreted as mature BDNF, (ii) reach the synaptic space unaltered as proBDNF and be processed into mature BDNF in the synaptic space, or (iii) be secreted without digestion [53]. A number of mechanisms affected by 66Met-BDNF can be hypothesized. First, 66Met-BDNF may alter the process of proBDNF modification with altered BDNF concentrations of immature and mature BDNF. Alternatively, this variant may lead to altered trafficking or might change receptor affinity. Chen et al. showed that 66Met-BDNF alters intracellular trafficking and proteolytic processing [54]. Finally, 66Met-BDNF might also influence the development of other neurons. Recently, Liao and colleagues showed that BDNF plays a role in directing the projection of TrkB neurons from the arcuate nucleus in the hypothalamus to the dorsomedial hypothalamic nucleus and paraventricular hypothalamic nucleus [55]. All these changes might affect hypothalamic functions including appetite and growth regulation.

We detected the rare frameshift variant rs539177035 (p.Cys34PhefsTer12) in a normal-weight boy with SNS. The variant was inherited from his normal-height, overweight father. The short and lean brother of the index patient also carried the variant. Additionally, we detected this variant in four of 789 analyzed fathers, three of whom transmitted this variant to their obese child. The two carrier girls were tall, while the male carrier had normal height. The deletion of two nucleotides (AT) affects *BDNF* exon VIII. BDNF uses four alternative translation start codons in different exons (I; VII, VIII, XI, see Fig. 1) that lead to four pre-pro BDNF isoforms [53]. p.Cys34PhefsTer12 is located at the N-terminal pre-hormone region and results in a translational termination after amino acid 44 with the loss of the longest transcript, pre-pro-BDNF (329aa). We can assume that this mutation has a functional relevance. It was previously suggested that the length of the pre-domain might affect intracellular BDNF trafficking with a preferred secretion of the immature isoform in the presence of the longer pre-domain versions [56]. ProBDNF and mature BDNF bind to their receptors p75 and TrkB with different affinities. While proBDNF has an important affinity to the p75 receptor, mature BDNF binds to the TrKB receptor [53]. The absence of the longest pre-pro-BDNF might thus result in altered trafficking of proBDNF and mature BDNF. These changes might lead to altered receptor activation with an increased or prolonged activation of TrkB. We checked the height development of the heterozygous father of the SNS child who was, at ascertainment, at a normal height. We discovered, by a questionnaire, that the father had shown symptoms for constitutional delay of growth and puberty (CDGP; short stature and delayed maturity) in his youth.

Finally, we found that the rare variant p.Val56Ala also affected *BDNF* exon VIII. This non-synonymous variant was detected in one child with SNS. The 56Ala-BDNF variant might alter the properties of the longest pre-pro-BDNF, so that trafficking and receptor activation are altered. Hence, in summary, our results cannot exclude or definitively support a relevance of BDNF variant Val56Ala for regulation of body height.

### No evidence for involvement of variants at the *LEP* or *MRAP2* genes in SNS

We did not detect variants in the coding region of *LEP*. Three synonymous variants in *MRAP2* were not associated with SNS. Hence, we conclude that variants in *LEP* and *MRAP2* do not have major effects on the SNS phenotype.

### Limitations

Limitations of this study include: (a) the diagnosis of idiopatic short stature [57] was not possible for all participants, so that the minimal consensus classification SNS [2] was used; (b) the comparison of the population-based data with our SNS sample, in that ExAC, EVS and KORA include a wide range of ages, weights and heights. A specifically matched young control group might show stronger association for the detected variants; (c) we could not analyse the genome-wide significant variants for adult height derived from the recent large-scale GWAS [3, 4].

## Conclusions

We analysed specific genes of major players of the leptinergic-melanocortinergic system in children and adolescents with SNS. We detected one rare *MC4R* mutation leading to partially reduced MC4R function, previously detected in individuals of normal height. Hence, a major relevance of this variant for SNS is unlikely. The detected *MC4R* polymorphism p.Val103Ile was nominally associated with SNS.

In *BDNF*, a novel non-synonymous variant and a rare frameshift variant, both affecting the centrally expressed longest preproBDNF transcript, were identified. Again, relevance for SNS is unlikely as persons with normal height also carry the mutation. We found association of the minor allele at the *BDNF* polymorphism p.Val66Met with SNS. GWAS in both children/adolescents and adults also revealed nominal associations of the minor allele with body height. In summary, our data point to an involvement of *MC4R* and *BDNF* polymorphisms with SNS, but do not support the involvement of rare mutations in *LEP*, *MC4R, MRAP2* or *BDNF* in our SNS children.

## Additional file

Additional file 1: **Table S1.** Primers, PCR conditions, fragment sizes and screening methods for *LEP*, *MC4R*, *MRAP2* and *BDNF* mutation screening [59–61]. **Table S2.** PCR-fragments and primers for *BDNF* expression analysis. **Table S3.** Association analyses. **Table S4:** Phenotype data of the index patients and available family members with infrequent variants in *BDNF* and *MC4R*. **Figure S1.** Amino acid sequence conservation in MC4R and BDNF among different species (including primates, rodents, laurasiatheria, placental mammals, sauropsia and fish). **Text S1.** Functional in vitro analyses for p.Met215Ile MC4R [62]. (DOCX 62 kb)

## Abbreviations

ANSD: Autonomic nervous system dysfunction
BDNF: Brain-derived neurotrophic factor
BMI: Body mass index
CCHS: Congenital central hypoventilation syndrome
CHOP: Children’s Hospital of Philadelphia
EC50: half maximal effective concentration
ERK: Extracellular-signal Regulated Kinase
ESP: Exome sequencing project
EVS: Exome variant server
ExAC: Exome Aggregation Consortium
GWAS: Genome-wide association study
HRM: High resolution melting
KORA: Cooperative Health Research in the Region Augsburg
LEP: Leptin
LTD: Long term depression
MC4R: Melanocortin 4 receptor
MRAP2: Melanocortin 2 receptor accessory protein 2
NDP-α-MSH: 4-L-Norleucin-7-D-Phenylalanin-α-Melanocyte-stimulating hormone
PCR: Polymerase chain reaction
SDS: Standard deviation score
SNP: Single nucleotide polymorphism
SNS: Short normal stature
SRE: Serum response element

## References

[1] Cohen P, Rogol AD, Deal CL, Saenger P, Reiter EO, Ross JL, Chernausek SD, Savage MO, Wit JM, Participants ICW (2008). Consensus statement on the diagnosis and treatment of children with idiopathic short stature: a summary of the growth hormone research society, the Lawson Wilkins pediatric Endocrine Society, and the European Society for Paediatric Endocrinology Workshop. J Clin Endocrinol Metab.

[2] Kranzler JH, Rosenbloom AL, Proctor B, Diamond FB, Watson M (2000). Is short stature a handicap? A comparison of the psychosocial functioning of referred and nonreferred children with normal short stature and children with normal stature. J Pediatr.

[3] Wood AR, Esko T, Yang J, Vedantam S, Pers TH, Gustafsson S, Chu AY, Estrada K, Luan J, Kutalik Z (2014). Defining the role of common variation in the genomic and biological architecture of adult human height. Nat Genet.

[4] Marouli E, Graff M, Medina-Gomez C, Lo KS, Wood AR, Kjaer TR, Fine RS, Lu Y, Schurmann C, Highland HM et al.. Rare and low-frequency coding variants alter human adult height. Nature. 2017. 10.1038/nature21039 PMC5302847 28146470

[5] Martinelli CE, Keogh JM, Greenfield JR, Henning E, van der Klaauw AA, Blackwood A, O'Rahilly S, Roelfsema F, Camacho-Hübner C, Pijl H (2011). Obesity due to melanocortin 4 receptor (MC4R) deficiency is associated with increased linear growth and final height, fasting hyperinsulinemia, and incompletely suppressed growth hormone secretion. J Clin Endocrinol Metab.

[6] Farooqi IS, Keogh JM, Yeo GS, Lank EJ, Cheetham T, O'Rahilly S (2003). Clinical spectrum of obesity and mutations in the melanocortin 4 receptor gene. N Engl J Med.

[7] Lango Allen H, Estrada K, Lettre G, Berndt SI, Weedon MN, Rivadeneira F, Willer CJ, Jackson AU, Vedantam S, Raychaudhuri S (2010). Hundreds of variants clustered in genomic loci and biological pathways affect human height. Nature.

[8] Asai M, Ramachandrappa S, Joachim M, Shen Y, Zhang R, Nuthalapati N, Ramanathan V, Strochlic DE, Ferket P, Linhart K (2013). Loss of function of the melanocortin 2 receptor accessory protein 2 is associated with mammalian obesity. Science.

[9] Rios M, Fan G, Fekete C, Kelly J, Bates B, Kuehn R, Lechan RM, Jaenisch R (2001). Conditional deletion of brain-derived neurotrophic factor in the postnatal brain leads to obesity and hyperactivity. Mol Endocrinol.

[10] Hebebrand J, Hinney A, Knoll N, Volckmar AL, Scherag A (2013). Molecular genetic aspects of weight regulation. Dtsch Arztebl Int.

[11] Ramachandrappa S, Farooqi IS (2011). Genetic approaches to understanding human obesity. J Clin Invest.

[12] Funcke JB, von Schnurbein J, Lennerz B, Lahr G, Debatin KM, Fischer-Posovszky P, Wabitsch M (2014). Monogenic forms of childhood obesity due to mutations in the leptin gene. Mol Cell Pediatr.

[13] Murray PG, Read A, Banerjee I, Whatmore AJ, Pritchard LE, Davies RA, Brennand J, White A, Ross RJ, Clayton PE (2011). Reduced appetite and body mass index with delayed puberty in a mother and son: association with a rare novel sequence variant in the leptin gene. Eur J Endocrinol.

[14] Hinney A, Volckmar AL, Knoll N (2013). Melanocortin-4 receptor in energy homeostasis and obesity pathogenesis. Prog Mol Biol Transl Sci.

[15] Wang D, Ma J, Zhang S, Hinney A, Hebebrand J, Wang Y, Wang HJ (2010). Association of the MC4R V103I polymorphism with obesity: a Chinese case-control study and meta-analysis in 55,195 individuals. Obesity (Silver Spring).

[16] Stutzmann F, Vatin V, Cauchi S, Morandi A, Jouret B, Landt O, Tounian P, Levy-Marchal C, Buzzetti R, Pinelli L (2007). Non-synonymous polymorphisms in melanocortin-4 receptor protect against obesity: the two facets of a Janus obesity gene. Hum Mol Genet.

[17] Loos RJ, Lindgren CM, Li S, Wheeler E, Zhao JH, Prokopenko I, Inouye M, Freathy RM, Attwood AP, Beckmann JS (2008). Common variants near MC4R are associated with fat mass, weight and risk of obesity. Nat Genet.

[18] Huszar D, Lynch CA, Fairchild-Huntress V, Dunmore JH, Fang Q, Berkemeier LR, Gu W, Kesterson RA, Boston BA, Cone RD (1997). Targeted disruption of the melanocortin-4 receptor results in obesity in mice. Cell.

[19] Aspiras AC, Rohner N, Martineau B, Borowsky RL, Tabin CJ (2015). Melanocortin 4 receptor mutations contribute to the adaptation of cavefish to nutrient-poor conditions. Proc Natl Acad Sci U S A.

[20] Zhang C, Forlano PM, Cone RD (2012). AgRP and POMC neurons are hypophysiotropic and coordinately regulate multiple endocrine axes in a larval teleost. Cell Metab.

[21] Schonnop L, Kleinau G, Herrfurth N, Volckmar AL, Cetindag C, Müller A, Peters T, Herpertz S, Antel J, Hebebrand J (2016). Decreased melanocortin-4 receptor function conferred by an infrequent variant at the human melanocortin receptor accessory protein 2 gene. Obesity (Silver Spring).

[22] Sebag JA, Zhang C, Hinkle PM, Bradshaw AM, Cone RD (2013). Developmental control of the melanocortin-4 receptor by MRAP2 proteins in zebrafish. Science.

[23] Xu B, Goulding EH, Zang K, Cepoi D, Cone RD, Jones KR, Tecott LH, Reichardt LF (2003). Brain-derived neurotrophic factor regulates energy balance downstream of melanocortin-4 receptor. Nat Neurosci.

[24] Elks CE, Loos RJ, Sharp SJ, Langenberg C, Ring SM, Timpson NJ, Ness AR, Davey Smith G, Dunger DB, Wareham NJ (2010). Genetic markers of adult obesity risk are associated with greater early infancy weight gain and growth. PLoS Med.

[25] Wudy SA, Hagemann S, Dempfle A, Ringler G, Blum WF, Berthold LD, Alzen G, Gortner L, Hebebrand J (2005). Children with idiopathic short stature are poor eaters and have decreased body mass index. Pediatrics.

[26] Dempfle A, Wudy SA, Saar K, Hagemann S, Friedel S, Scherag A, Berthold LD, Alzen G, Gortner L, Blum WF (2006). Evidence for involvement of the vitamin D receptor gene in idiopathic short stature via a genome-wide linkage study and subsequent association studies. Hum Mol Genet.

[27] Heid IM, Vollmert C, Hinney A, Döring A, Geller F, Löwel H, Wichmann HE, Illig T, Hebebrand J, Kronenberg F (2005). Association of the 103I MC4R allele with decreased body mass in 7937 participants of two population based surveys. J Med Genet.

[28] Hinney A, Hohmann S, Geller F, Vogel C, Hess C, Wermter AK, Brokamp B, Goldschmidt H, Siegfried W, Remschmidt H (2003). Melanocortin-4 receptor gene: case-control study and transmission disequilibrium test confirm that functionally relevant mutations are compatible with a major gene effect for extreme obesity. J Clin Endocrinol Metab.

[29] Wittwer CT, Reed GH, Gundry CN, Vandersteen JG, Pryor RJ (2003). High-resolution genotyping by amplicon melting analysis using LCGreen. Clin Chem.

[30] Arenas-Hernandez M, Vega-Sanchez R (2013). Housekeeping gene expression stability in reproductive tissues after mitogen stimulation. BMC Res Notes.

[31] Tao YX (2014). Constitutive activity in melanocortin-4 receptor: biased signaling of inverse agonists. Adv Pharmacol.

[32] Xiang Z, Proneth B, Dirain ML, Litherland SA, Haskell-Luevano C (2010). Pharmacological characterization of 30 human melanocortin-4 receptor polymorphisms with the endogenous proopiomelanocortin-derived agonists, synthetic agonists, and the endogenous agouti-related protein antagonist. Biochemistry.

[33] Zhao J, Li M, Bradfield JP, Zhang H, Mentch FD, Wang K, Sleiman PM, Kim CE, Glessner JT, Hou C (2010). The role of height-associated loci identified in genome wide association studies in the determination of pediatric stature. BMC Med Genet.

[34] Marshall WA, Tanner JM (1970). Variations in the pattern of pubertal changes in boys. Arch Dis Child.

[35] Stevens RC, Chook YM, Cho CY, Lipscomb WN, Kantrowitz ER (1991). Escherichia coli aspartate carbamoyltransferase: the probing of crystal structure analysis via site-specific mutagenesis. Protein Eng.

[36] Szczepek M, Beyrière F, Hofmann KP, Elgeti M, Kazmin R, Rose A, Bartl FJ, von Stetten D, Heck M, Sommer ME (2014). Crystal structure of a common GPCR-binding interface for G protein and arrestin. Nat Commun.

[37] Gu W, Tu Z, Kleyn PW, Kissebah A, Duprat L, Lee J, Chin W, Maruti S, Deng N, Fisher SL (1999). Identification and functional analysis of novel human melanocortin-4 receptor variants. Diabetes.

[38] Hinney A, Bettecken T, Tarnow P, Brumm H, Reichwald K, Lichtner P, Scherag A, Nguyen TT, Schlumberger P, Rief W (2006). Prevalence, spectrum, and functional characterization of melanocortin-4 receptor gene mutations in a representative population-based sample and obese adults from Germany. J Clin Endocrinol Metab.

[39] Xiang Z, Litherland SA, Sorensen NB, Proneth B, Wood MS, Shaw AM, Millard WJ, Haskell-Luevano C (2006). Pharmacological characterization of 40 human melanocortin-4 receptor polymorphisms with the endogenous proopiomelanocortin-derived agonists and the agouti-related protein (AGRP) antagonist. Biochemistry.

[40] Weese-Mayer DE, Bolk S, Silvestri JM, Chakravarti A (2002). Idiopathic congenital central hypoventilation syndrome: evaluation of brain-derived neurotrophic factor genomic DNA sequence variation. Am J Med Genet.

[41] Marcus CL, Jansen MT, Poulsen MK, Keens SE, Nield TA, Lipsker LE, Keens TG (1991). Medical and psychosocial outcome of children with congenital central hypoventilation syndrome. J Pediatr.

[42] Friedel S, Horro FF, Wermter AK, Geller F, Dempfle A, Reichwald K, Smidt J, Brönner G, Konrad K, Herpertz-Dahlmann B (2005). Mutation screen of the brain derived neurotrophic factor gene (BDNF): identification of several genetic variants and association studies in patients with obesity, eating disorders, and attention-deficit/hyperactivity disorder. Am J Med Genet B Neuropsychiatr Genet.

[43] Zegers D, Hendrickx R, Verrijken A, Van Hoorenbeeck K, Van Camp JK, de Craemer V, Rooman RP, Desager KN, Massa G, Van Gaal LF (2014). Screening for genetic variants in BDNF that contribute to childhood obesity. Pediatr Obes.

[44] Hall D, Dhilla A, Charalambous A, Gogos JA, Karayiorgou M (2003). Sequence variants of the brain-derived neurotrophic factor (BDNF) gene are strongly associated with obsessive-compulsive disorder. Am J Hum Genet.

[45] Geller B, Badner JA, Tillman R, Christian SL, Bolhofner K, Cook EH (2004). Linkage disequilibrium of the brain-derived neurotrophic factor Val66Met polymorphism in children with a prepubertal and early adolescent bipolar disorder phenotype. Am J Psychiatry.

[46] Toda T, Momose Y, Murata M, Tamiya G, Yamamoto M, Hattori N, Inoko H (2003). Toward identification of susceptibility genes for sporadic Parkinson's disease. J Neurol.

[47] Ribasés M, Gratacòs M, Fernández-Aranda F, Bellodi L, Boni C, Anderluh M, Cavallini MC, Cellini E, Di Bella D, Erzegovesi S (2004). Association of BDNF with anorexia, bulimia and age of onset of weight loss in six European populations. Hum Mol Genet.

[48] Ribasés M, Gratacòs M, Armengol L, de Cid R, Badía A, Jiménez L, Solano R, Vallejo J, Fernández F, Estivill X (2003). Met66 in the brain-derived neurotrophic factor (BDNF) precursor is associated with anorexia nervosa restrictive type. Mol Psychiatry.

[49] Mössner R, Walitza S, Lesch KP, Geller F, Barth N, Remschmidt H, Hahn F, Herpertz-Dahlmann B, Fleischhaker C, Schulz E (2005). Brain-derived neurotrophic factor V66M polymorphism in childhood-onset obsessive-compulsive disorder. Int J Neuropsychopharmacol.

[50] Wendland JR, Kruse MR, Cromer KR, Cromer KC, Murphy DL (2007). A large case-control study of common functional SLC6A4 and BDNF variants in obsessive-compulsive disorder. Neuropsychopharmacology.

[51] Mizui T, Ishikawa Y, Kumanogoh H, Lume M, Matsumoto T, Hara T, Yamawaki S, Takahashi M, Shiosaka S, Itami C (2015). BDNF pro-peptide actions facilitate hippocampal LTD and are altered by the common BDNF polymorphism Val66Met. Proc Natl Acad Sci U S A.

[52] Egan MF, Kojima M, Callicott JH, Goldberg TE, Kolachana BS, Bertolino A, Zaitsev E, Gold B, Goldman D, Dean M (2003). The BDNF val66met polymorphism affects activity-dependent secretion of BDNF and human memory and hippocampal function. Cell.

[53] Martínez-Levy GA, Cruz-Fuentes CS (2014). Genetic and epigenetic regulation of the brain-derived neurotrophic factor in the central nervous system. Yale J Biol Med.

[54] Chen ZY, Patel PD, Sant G, Meng CX, Teng KK, Hempstead BL, Lee FS (2004). Variant brain-derived neurotrophic factor (BDNF) (Met66) alters the intracellular trafficking and activity-dependent secretion of wild-type BDNF in neurosecretory cells and cortical neurons. J Neurosci.

[55] Liao GY, Bouyer K, Kamitakahara A, Sahibzada N, Wang CH, Rutlin M, Simerly RB, Xu B (2015). Brain-derived neurotrophic factor is required for axonal growth of selective groups of neurons in the arcuate nucleus. Mol Metab.

[56] Aid T, Kazantseva A, Piirsoo M, Palm K, Timmusk T (2007). Mouse and rat BDNF gene structure and expression revisited. J Neurosci Res.

[57] Ranke MB (1996). Towards a consensus on the definition of idiopathic short stature. Horm Res.

[58] Tarnow P, Rediger A, Brumm H, Ambrugger P, Rettenbacher E, Widhalm K, Hinney A, Kleinau G, Schaefer M, Hebebrand J (2008). A heterozygous mutation in the third transmembrane domain causes a dominant-negative effect on signalling capability of the MC4R. Obes Facts.

[59] Hinney A, Schmidt A, Nottebom K, Heibült O, Becker I, Ziegler A (1999). Several mutations in the melanocortin-4 receptor gene including a nonsense and a frameshift mutation associated with dominantly inherited obesity in humans. J Clin Endocrinol Metab.

[60] Gotoda T, Scott J, Aitman TJ (1997). Molecular screening of the human melanocortin-4 receptor gene: identification of a missense variant showing no association with obesity, plasma glucose, or insulin. Diabetologia.

[61] Hebebrand J, Geller F, Dempfle A, Heinzel-Gutenbrunner M, Raab M, Gerber G (2004). Binge-eating episodes are not characteristic of carriers of melanocortin-4 receptor gene mutations. Mol Psychiatry.

[62] Tarnow P, Rediger A, Schulz A, Grüters A, Biebermann H (2012). Identification of the translation start site of the human melanocortin 3 receptor. Obes Facts.

