# Supplementary material for: Relevance of polymorphisms in MC4R and BDNF in short normal stature
Source: BMC Pediatr. 2018 Aug 22;18:278. doi: 10.1186/s12887-018-1245-1 (PMC6106737; doi:10.1186/s12887-018-1245-1)
Supplement: Supplementary file 1 — Table S1. Primers, PCR conditions, fragment sizes and screening methods for LEP, MC4R, MRAP2 and BDNF mutation screening [59–61]. Table S2. PCR-fragments and primers for BDNF expression analysis. Table S3. Association analyses. Table S4: Phenotype data of the index patients and available family members with infrequent variants in BDNF and MC4R. Figure S1. Amino acid sequence conservation in MC4R and BDNF among different species (including primates, rodents, laurasiatheria, placental mammals, sauropsia and fish). Text S1. Functional in vitro analyses for p.Met215Ile MC4R [62]. (DOCX 62 kb) [file 12887_2018_1245_MOESM1_ESM.docx]

| **gene** | **fragment** | **name** | **primer** | **GC-content (%)** | **size (bp)** | **screening -method** |
| --- | --- | --- | --- | --- | --- | --- |
| *LEP* | F1 | OB1AB F1F | TCTGAGATACCGGCTCCTTG | 55 | 297 | dHPLC |
|  |  | OB1AB F1R | GACTATCTGGGTCCAGTGCC | 60 |  |  |
|  | F2 | OB2AB F2F | GATTCCTCCCACATGCTGAG | 55 | 496 |  |
|  |  | OB2AB F2R | TGCAATGCTCTTCAATCCTG | 45 |  |  |
| *MC4R* | F1 | MC4R F1 | ATCAATTCAGGGGGACACTG | 50 | 615 | dHPLC |
|  |  | MC4R R1 | GACAGCACTACTATCTGAGT | 45 |  |  |
|  | F2 | MC4R F2 | ATGCTCTCCAGTACCATAACA | 43 | 622 |  |
|  |  | MC4R R2 | TGCAGAAGTACAATATTCAGG | 38 |  |  |
|  | F3 | MC4R F3 | GCCCAAGATTTTAAAGTGATGAT | 35 | 458 |  |
|  |  | MC4R R3 | GGTGAATGCAGATTCTTGT | 42 |  |  |
| *MRAP2* | F1 | MRAP2 F1-F | CTGCCAAACTCCTGAATGCG | 55 | 311 | dHPLC |
|  |  | MRAP2 F1-R | CAGGAGAAGTAATCCTTGGGG | 52 |  |  |
|  | F2 | MRAP2 F2-F | GCCTCTCCAAAAAGCTCTGA | 50 | 235 | HRM |
|  |  | MRAP2 F2-R | TCCTCAGTCTCCTGTGAGGC | 60 |  |  |
|  | F3 | MRAP2 F3-F | GAATGGGCTGGAGTGTAAGC | 55 | 581 | Sanger re-sequencing |
|  |  | MRAP2 F4-R | AAAGGGGTCTCTTCTCTGCC | 55 |  |  |
| *BDNF* | F1 | BDNF F2F | CCACATGGCTCATTATGCAC | 50 | 595 | Sanger re-sequencing |
|  |  | BDNF F2R | CAGGTGATGGAAGAAACTGGA | 48 |  |  |
|  | F2 | BDNF F3F | CGGTGAAAGAAAGCCCTAAC | 50 | 1006 |  |
|  |  | BDNF F3R | GTTTCCCTTCTGGTCATGGA | 50 |  |  |

**Table S1: Primers, PCR conditions, fragment size and screening method for *LEP*, *MC4R*, *MRAP2* and *BDNF* mutation screens**

We divided *LEP* in two fragments, *MC4R* and *MRAP2* in three fragments and *BDNF* in two fragments. Primer design was performed with gene distiller (http://www.genedistiller.org)

Two PCR fragments, flanking translated exons II and III, were used to screen the coding region of *LEP* (GRCh37/hg19: chr7:127,881,331-127,897,682). The protein-coding exon of *MC4R* (GRCh37/hg19: chr18:58,038,564-58,040,001) was screened with overlapping fragments [59]. The three coding exons (II-IV) of *MRAP2* (GRCh37/hg19: chr6:84,743,420-84,800,605) were amplified with three PCR fragments [21]. In contrast to Schonnop et al. fragment 3 and 4 were screened within one fragment (see S1 Table). The coding exon IX of *BDNF* (GRCh37/hg19: chr11:27,676,442-27,722,600) encoding pro-BDNF was analyzed with one PCR fragment. Additionally exon VIII was screened with one PCR fragment encoding for the longest BDNF pre-domain. For details pertaining to fragment length, primer sequence and screening method see S1 Table.

The screen by denaturing high performance liquid chromatography (dHPLC) was performed as described previously [28]. HRM is a post-PCR analysis method based on the detection of different melting temperatures using SYBR® Green a florescent DNA dye. Commercial re-sequencing war performed by LGC Genomics (Berlin, Germany). Samples with aberrant dHPLC or HRM patterns were also re-sequenced commercially (Seq Lab, Göttingen, Germany). Val103Ile, Ile251Leu of *MC4R* were genotyped with PCR-RFLP [60, 61]. Taqman-genotyping of the GWAS height SNP rs17782313 near *MC4R* was performed with the TaqMan SNP Genotyping Assay (Applied Biosystems assay: C_32667060). For validity all results were controlled by two experienced individuals. Discrepancies were solved by consensus or re-typing

**Table S2: PCR-fragments and primer for the BDNF expression analysis:**

| **name** | **primer** | **size (bp)** |
| --- | --- | --- |
| GAPDH forward* | AGCCACATCGCTCAGACAC | 65 |
| GAPDH reverse* | GCCCAATACGACCAAATCC |  |
| BDNF008 F1F | AGGTCCACACATGTTTTGGC | 247 |
| BDNF008 F1R | CCTTGTCCTCGGATGTTTGC |  |
| BDNFIX F1F | AACATCCGAGGACAAGGTGG | 220 |
| BDNFIX F1R | TACTGAGCATCACCCTGGAC |  |

* Primer adapted from [30].

**Table S3: Association analysis**

| ***gene***  **variant** | **calculation** | **SNS: ExAC (60,675-60,699 with different ethnic background)**  **34,000 European descent** | **SNS: KORA (n=7,937)** | **SNS: EVS (n=4,300)** |
| --- | --- | --- | --- | --- |
| *MC4R*  Ile251Leu | variant carrier frequency (%) | (2.16: 1.36)  2.16: 2.00 | NA | 2.16 : 2.33 |
|  | P-value | (0.35)  0.87 | NA | 0.885 |
| *MC4R*  Val103Ile | variant carrier frequency (%) | (6.49: 3.34)  6.49: 3.86 | 6.49 : 3.67 | 6.49 : 3.70 |
|  | P-value | (0.023)  0.065 | 0.045 | 0.052 |
| *BDNF*  Val66Met | allele frequency (%) | (23.51: 19.37)  23.51: 19.26 | NA | 23.51 : 19.07 |
|  | P-value | (0.044)  0.040 | NA | 0.034 |

NA: not available; SNS: short normal stature; ExAC: The Exome Aggregation Consortium; EVS: Exome variant server, two-sided χ2-tests were used and nominal p-values are given

**Table S4: Phenotype data of the index patients and available family members with infrequent variants in BDNF and MC4R**

| **Gene** | **AA-exchange*** | **Family member** | **Sex** | **Age (years)** | **Height (cm)** | **Height SDS** | **Height perc.** | **Weight (kg)** | **BMI (kg/m²)** | **BMI SDS** | **BMI perc.** | **Tanner stage (ref)** | **Bone age**  **(years)** | **Leptin (µg/L) [SDS]** |
| --- | --- | --- | --- | --- | --- | --- | --- | --- | --- | --- | --- | --- | --- | --- |
| ***MC4R*** | Met251Ile | Index | m | 11.13 | 129.8 | -2.33 | 0.98 | 27.4 | 16.26 | -0.58 | 28.14 | 1 (0-12) | - 1.55 | 1.33  [0.21] |
|  | WT | Mother | f | 34 | 163.5 |  |  | 76.7 | 28.69 |  | 85.23 |  |  |  |
|  | NA | Father | m | 35 | 164 |  |  | 75 | 27.89 |  | 71.86 |  |  |  |
|  | WT | Sibling | m | 14 | 158 | -1.12 | 13.23 | 53.2 | 21.28 |  | 73.73 | 2 (9.5-14.5) | NA | 1.39  [-2.29] |
|  | Thr112Met | Index | m | 15.16 | 148.8 | -3.14 | 0.08 | 41.1 | 18.66 | -0.49 | 31.19 | 3 (10.1-16.5) | - 2.28 | 1.67 [0.97] |
|  | Thr112Met | Index | f | 17.58 | 155.1 | -1.98 | 2.39 | 51.8 | 21.53 | 0.15 | 55.85 | 4 (10.4-15.3) | - 0.27 | 6.4  [-0.90] |
| ***BDNF*** | Thr2Ile | Index | m | 11.08 | 131.2 | -2.11 | 1.79 | 30.2 | 17.54 | 0.04 | 51.42 | 1 (0-12) | - 0.57 | 5.02 [2.03] |
|  | Thr2Ile | Mother | f | 36 | 157.5 |  |  | 60.3 | 24.31 |  | 57.33 |  |  |  |
|  | WT | Father | m |  | 168.8 |  |  | 83.1 | 29.16 |  | 76.10 |  |  |  |
|  | Thr2Ile | Sibling | m | 4 | 100 | -1.94 | 2.62 | 15.2 | 15.2 | 0.15 | 44.00 | 1 (0-12) | NA | 2.16 [1.64] |
|  | Cys34PhefsTer12 | Index | m | 10.33 | 130.1 | -1.87 | 3.09 | 30.6 | 18.08 | 0.42 | 66.18 | 2 (9.5-14.5) | - 3 | 1.74  [-0.21] |
|  | WT | Mother | f | 32 | 154.2 |  |  | 48.7 | 20.48 |  | 19.50 |  |  |  |
|  | Cys34PhefsTer12 | Father | m | 33 | 173.2 |  |  | 93.6 | 31.2 |  | 90.71 |  |  |  |
|  | Cys34PhefsTer12 | Sibling | m | 7 | 118.3 | -1.34 | 8.97 | 22.7 | 16.2 | 0.27 | 60.68 | 1 (0-12) | NA | 1.49 [0.44] |
|  | Val56Ala | Index | m | 14,58 | 142.0 | -3.40 | 0.03 | 36.1 | 17.90 | -0.72 | 23.49 | NA | NA | NA |
|  | Val56Ala | Mother | f | NA |  |  |  |  |  |  |  |  |  |  |

AA: amino acid; all variants were detected heterozygously, WT: homozygous wild type; NA: not available

**Figure S1: Amino acid sequence conservation in MC4R and BDNF among different species (including primates, rodents, laurasiatheria, placental mammals, sauropsia, fish)**

**MC4R**  Val103Ile Thr112Met Met 215Ile Ile251Leu

**Primates**

Homo sapiens NGSETIVITLLNSTDTDAQSFTVN TMFFTMLALMASLYVHMFLMARLHIKRIAVLPGTGAIRQGANMKGAITLTILIGVFV

Gorilla gorilla gorilla NGSETIVITLLNSTDTDAQSFTVN TMFFTMLALMASLYVHMFLMARLHIKRIAVLPGTGAIRQGANMKGAITLTILIGVFV

Pan troglodytes NGSETIVITLLNGTDTDAQSFTVN TMFFTMLALMASLYVHMFLMARLHIKRIAVLPGTGAIRQGANMKGAITLTILIGVFV

Pongo abelii NGSETIVITLLNSTDTDAQSFTVN TMFFTMLALMASLYVHMFLMARLHIKRIAVLPGTGAIRQGANMKGAITLTILIGVFV

Nomascus leucogenys NGSETIVITLLNSTDTDAQSFTVN TMFFTMLALMASLYVHMFLMARLHIKRIAVLPGTGAIRQGANMKGAITLTILIGVFV

Chlorocebus sabaeus NGSETIVITLLNSTDTDTQSFTVN TMFFTMLALMASLYVHMFLMARLHIKRIAVLPGTGAIRQGANMKGAITLTILIGVFV

Papio Anubis NGSETIVITLLNSTDTDTQSFTVN TMFFTMLALMASLYVHMFLMARLHIKRIAVLPGTGAIRQGANMKGAITLTILIGVFV

Macaca mulatta NGSETIVITLLNSTDTDTQSFTVN TMFFTMLALMASLYVHMFLMARLHIKRIAVLPGTGAIRQGANMKGAITLTILIGVFV

Callithrix jacchus NGSETIVITLLNSTDTDAQSFTVN TMFFTMLALMASLYVHMFLMARLHIKRIAVLPGTGAIRQGANMKGAITLTILIGVFV

Otolemur garnettii NGSETIVITLLNSTDTDAQSFTVN TMFLTMLALMASLYVHMFLMARLHIKRIAVLPGTGAIRQGANMKGAITLTILIGVFV

Tarsius syrichta NGSETIVITLLNSTDTDAQSFTVN TMFFTMLALMASLYVHMFLMARLHIKRIAVLPGTGTIHQGANMKGAITLTILIGVFV

**Rodents**

Rattus norvegicus NGSETIVITLLNSTDTDAQSFTVN TMFFTMLVLMASLYVHMFLMARLHIKRIAVLPGTGTIRQGANMKGAITLTILIGVFV

Mus musculus NGSETIVITLLNSTDTDAQSFTVN SMFFTMLVLMASLYVHMFLMARLHIKRIAVLPGTGTIRQGTNMKGAITLTILIGVFV

Tupaia belangeri NGSETIVITLLNSTDTDAQSFTVN TMFFTMLALMASLYVHMFLMARLHIKRIAVLPGTGTIRQGANMKGAITLTILIGVFV

Oryctolagus cuniculus NGSETIVITLLNSTDTDAQSFTVN TMFFTMLALMASLYVHMFLMARLHIKRIAVLPGTGAIRQGANMKGAITLTILIGVFV

Cavia porcellus NGSETIVITLLNSTDTDAQSFTVN TMFFTMLALMASLYVHMFLMARLHIKRIAVLPGTGTIRQGANMKGAITLTILIGVFV

Ictidomys tridecemlineatus NGSETIVITLLNSTDTDAQSFTVN TMFFTMLALMASLYVHMFLMARLHIKRIAVLPGTGAIRQGANMKGAITLTILIGVFV

**Laurasiatheria**

Sus scrofa NGSETIVITLLNSTDTDAQSFTVN TVFFTMLALMASLYVHMFLMARLHIKRIAVLPGTGTIRQGANMKGAITLTILIGVFV

Pteropus vampyrus NGSETIVITLLNSTDTDAQSFTVN TMFFTMLALMASLYVHMFLMARLHIKRIAVLPGTGTIRQGANMKGAITLTILIGVFV

Vicugna pacos NGSETIVITLLNSTDTDTQSFTVN TMFFTMLALMASLYVHMFLMARLHIKRIAVLPGTGTVRQGANMKGAITLTILIGVFV

Equus caballus NGSETIVITLLNSTDTDAQSFTVN TMFFTMLALMASLYVHMFLMARLHIKRIAVLPGTGTIRQGANMKGAITLTILIGVFV

Tursiops truncatus NGSETIVITLLNSTDTDAQGFTVN TVFFTMLALMASLYVHMFLMARLHIKRIAVLPGTGAVRQGANMKGAITLTILIGVFV

Ovis aries NRSETIVITLLNSTDTDAQSFTVN TVFFTMLALMASLYVHMFLMARLHIKRIAVLPGTGAIRQGANMKGAITLTILIGVFV

Felis catus NGSETIVITLLNSTDTDAQSFTVN TMFFTMLALMASLYVHMFLMARLHIKRIAVLPGTGTIRQGANMKGAITLTILIGVFV

Canis lupus familiaris NGSETIVITLLNSTDTDAQSFTVN TMFFTMLALMASLYVHMFLMARLHIKRIAVLPGTGTIRQGANMKGAITLTILIGVFV

Bos Taurus NGSETIVITLLNSTDTDAQSFTVD TVFFTMLALMASLYVHMFLMARLHIKRIAVLPGSGTIRQGANMKGAITLTILIGVFV

Erinaceus europaeus NGSETIVITLLNSTETDAQSFTVN TMFFTMLALMASLYVHMFLMARLHIKRIAVLPGTGPIHQGANMKGAITLTILIGVFV

Ailuropoda melanoleuca NGSETIVITLLNSTDTDTQSFTVN TMFFTMLALMASLYVHMFLMARLHIKRIAVLPGTGTIRQGANMKGAITLTILIGVFV

Mustela putorius furo NGSETIVITLLNSTDTDAQSFTVN TMFFTMLALMASLYVHMFLMARLHIKRIAVLPGSGAIRQGANMKGAITLTILIGVFV

Myotis lucifugus NGTETIAIILLNGTDTKAPSFSEN SVFLTMLALMASLYVHMFLMARLHMKRIAVLPGTGTIRQGANMKGAITLTILIGVFV

**Other placental mammals**

Dasypus novemcinctus NGSETIVIILLNSTDSDAQSFTVN TMFFTMLALMASLYVHMFLMARLHIKRIAVLPGPGAIRPGANMKGAITLTILIGVFV

Loxodonta Africana NGSETIVITLLNSTDTDAQSFTVN AMFFTMLALMASLYVHMFLMARLHIKRIAVLPGTGTIRQGANMKGAITLTILIGVFV

Choloepus hoffmanni NGSETIVITLXXXXXXXXXXXXXX TMFFTMLALMASLYVHMFLMARLHIKRIAVLPGTGAVRQGANMKGAITLTILIGVFV

**Sauropsia**

Anolis carolinensis NGSETIVITLLNNTDVGGHSFTVN SMFFTMLVLMASLYVHMFLLARLHIKKIAILPGTGPICQRANMKGAITLTILIGVFV

Pelodiscus sinensis NGSETIVITLLNNTDTGAQSFTVN SMFFTMLVLMASLYVHMFMLARLHIKKIAVLPGTGTIRQGANMKGAITLTILIGVFV

Meleagris gallopavo NGSETIVITLLNNTDTDAQSFTIN SMFFTMLILMASLYVHMFMMARMHIKKIAVLPGTGPIRQGANMKGAITLTILIGVFV

Anas platyrhynchos NGSETIVITLLNNTDTDAQSFTIN SMFFTMLILMASLYVHMFMMARMHIKKIAVLPGTGPIRQGANMKGAITLTILIGVFV

Ficedula albicollis NGSETIVITLLNNTDTDAQSFTIN SMFFTMLILMASLYVHMFMMARMHIKKIAVLPGSGPVRQGANMKGAITLTILIGVFV

Gallus gallus NGSETIVITLLNNTDTDAQSFTIN SMFFTMLILMASLYVHMFMMARMHIKKIAVLPGTGPIRQGANMKGAITLTILIGVFV

Taeniopygia guttata NGSETIVITLLNNTDTDAQSFTIN SMFFTMLILMASLYVHMFMMARMHIKKIAVLPGTGPVRQGANMKGAITLTILIGVFV

**Fish**

Poecilia Formosa NASETIVIALINGGSLTIPVTLIK TMFFTMLVLMASLYVHMFLLARLHMKRIAALPGNAPIQQRANMKGAITLTILLGVFV

Astyanax mexicanus NSTETVVMALITGGNLSISGGVAK SMFFAMLALMASLYVHMFLLARLHMKRIAALPGNGPVPQAANMKGAVTLTILLGVFV

Gadus morhua NASETIVISLINSGSLPIPVTLIK TMFLTMLVLMASLYVHMFLLARLHMKRIAALPGNAPIHQRANMKGAITLTILLGVFV

Takifugu rubripes NASETIVIALINSGTLTIPATLIK TMFFTMLVLMASLYVHMFLLARLHMKRIAAMPGNAPIHQRANLKGAITLTILLGVFV

Oryzias latipes NASETIVIALINGGNLSIPVRLIK TMFFTMLVLMASLYVHMFLLARLHMKRIAALPGNAPIHQRANMKGAITLTILLGVFV

Lepisosteus oculatus NAWETIVMALITSGHLTIQDNLIK TMFFTMLALMASLYVHMFMLARLHMKRIAALPGNGTIHQGANMKGAITLTILLGVFV

Gasterosteus aculeatus NASETIVIALIAGGTLTIPVALIR TMFFTMLVLMASLYVHMFLLARLHIKRIAALPGHAPIHQRANMKGAITLTILLGVFV

Oreochromis niloticus NASETIVIALINGGSLTIPVTLIK TMFFTMLVLMASLYVHMFLLARLHMKRIAALPGNAPIQQRANMKGAITLTILLGVFV

Tetraodon nigroviridis NASETIVIALINGGTLTIPARLIK TTFFTMLVLMASLYVHMFLLARLHMKRIAAMPGNAPIHQRANMKGAITLTILLGVFV

Danio rerio NASETVVMALITGGNLTNRESIIK SMFFTMLALMASLYVHMFLLARLHMKRIAALPGNGPIWQAANMKGAITITILLGVFV

**Others (e.g. invertebrates)**

Macropus eugenii NGSETIVITLLNSTDTDAQSFTVN SMFFTMLALMASLYVHMFLMARLHIKRIAVLPGTGAIRQGANMKGAITLTILIGVFV

Monodelphis domestica NGSETIVITLLNSTDTDAQSFTVN TMFFTMLALMASLYVHMFLMARLHIKRIAVLPGTGTIRQGANMKGAITLTILIGVFV

Sarcophilus harrisii NGSETIVITLLNSTETDAQSFTVN SMFFTMLALMASLYVHMFLMARLHIKRIAVLPGTGTIRQGANMKGAITLTILIGVFV

Ornithorhynchus anatinus NGSETIVITLLNATDGGAAQSFAV ATFFAMLALMASLYVHMFLLARLHVKRIAGLPGPGAVRQGASMKGAVTLTILIGVFV

Latimeria chalumnae NACEAIAIALINDSYIMTADNFVK TMFFIMLVLIASLYVHMFMLARQHMKRIAALPGNGTVRQVANMKGAITLTILLGVFI

Ciona intestinalis FVFFHTMVILSCPNHFSCQNSSVQ SLTAAVVVIALNIWMVKFAISIIMSMQTPP--STQIGIINESLKPATTLALFVVSFI

**BDNF** Val56Ala Thr2Ile Val66Met

**Primates**

Homo sapiens HTCFGVYPHASVWHDCASQKKGCAVYLHV SVEFNKLIPENGFIKFHQVRRVMTILFLTMVISY THGTLESVNGPKAGSRGLTSLADTFEHVIEELLDE

Gorilla gorilla gorilla HTCFGVYPQASVWHDCASQKKGCAVYLHV ----------------------MTILFLTMVISY THGTLESVNGPKAGSRGLTSLADTFEHVIEELLDE

Pan troglodytes ----------------------------- ----------------------MTILFLTMVISY THGTLESVNGPKAGSRGLTSLADTFEHVIEELLDE

Pongo abelii ----------------------------- SVEFNKLIPENGFIKFHQVRRVMTILFLTMVISY THGTLESVNGPKAGSRGLTSLADTFEHVIEELLDE

Nomascus leucogenys HTCFGVYPHASVWHDCASQKKGCAVYLHV SVEFNKLIPENGFIKFHQVRRVMTILFLTMVISY THGTLESVNGPKAGSRGLTSLADTFEHVIEELLDE

Macaca mulatta ----------------------------- ----------------------MTILFLTMVISY THGTLESVNGPKAGSRGLTSLADTFEHVIEELLDE

Callithrix jacchus HTCFGVYPHTSVWHDCASQKKGCAVCLRV SVEFNKPIPENGFIKFHQVRRVMTILFLTMVISY THGTLESVNGPKAGSRSLTSLADTFEHVIEELLDE

Otolemur garnettii ----------------------------- ---------------FHQVRRVMTILFLTMVISY THGTLESVNGPKAGSRGLTSLADTFEHVIEELLDE

Papio Anubis GTRAGRWSQNRN-HDVTPPPGTR------ --------ELCVDPEFHQVRRVMTILFLTMVISY THGTLESVNGPKAGSRGLTSLADTFEHVIEELLDE

Tarsius syrichta ----------------------------- ---------------FHQVRRVMTILFLTMVISY THGTLESMNGPKAGSRGLTALADTFEHVLEELLDE

Chlorocebus sabaeus ----------------------------- ----------------------MTILFLTMVISY THGTLESVNGPKAGSRGLTSLADTFEHVIEELLDE

**Rodents**

Oryctolagus cuniculus ----------------------------- ---------------FHQVRRVMTILFLTMVISY THGTLESVNGPKTGSRGLTSLADTFEHVIEELLDE

Ochotona princeps ----------------------------- -------MQSREEKWFHQVRRVMTILFLTMVISY THGTLESMNGPKTGSRGVTSLADTFEHVIEELLDE

Cavia porcellus ----------------------------- ----------------------MTILFLTMVISY THGALESATGPKVGARGLTSLADTFEHVIEELLVE

Ictidomys tridecemlineatus HTCFGIYPHTSVWHDYASQKKGCGVL--A SVEWNRLITENGLIKFHQVRRVMTILFLTMVISY THGTLESVNGPKAGSRGLTSLADTFEHVIEELLDE

Tupaia belangeri ----------------------------- ---------------FHQVRRVMTILFLTMVISY THGTLESGNGPQAGARGLTSLADTFEHVIEELLDE

Rattus norvegicus ----------------------------- ----------------------MTILFLTMVISY THGTLESVNGPRAGSRGLTSLADTFEHVIEELLDE

Mus musculus ----------------------------- --------------MFHQVRRVMTILFLTMVISY THGTLESVNGPRAGSRGLTSLADTFEHVIEELLDE

**Laurasiatheria**

Sus scrofa ----------------------------- -----------------------------MVISY THGTLESVNGPKAGSRGLTSLADTFEHVIEELLDE

Pteropus vampyrus -----MQSRKEKW---------------- ---------------FHQVRRVMTILFLTMVISY THGTLESVNGPKAGSRGLTALADTFEHVIEELLDE

Vicugna pacos QSWKGEW---------------------- ---------------FHQVRRVMTILFLTMVISY THGTLESVNGPKAGSRGLTSLADTFEHVIEELLDE

Equus caballus ----------------------------- ---------------IHQVRRVMTILFLTMVISY THGTLESVNGPKAGSRGLTSLADTFEHVIEDLLDE

Sorex araneus ----------------------------- ---------------FHQVRRVMTILFLTMVISY THGTLESVNGPQAGSRGLTSLADTFEHVIEELLDE

Tursiops truncatus ----------------------------- ---------------FHQVRRVMTILFLTMVISY THGTLESVNGPKAGSRGLTSLADTVEHVIEELLDE

Ovis aries ----------------------------- ----------------------MTILFLTMVISY THGTLESMNGPKVGSRGLTSLADTFEHVIEELLDE

Felis catus ----------------------------- ----------------------MTILFLTMVISY THGTLESVNGPKAGSRGLTSLADTFEHVIEELLDE

Canis lupus familiaris HTRLGSCPHASSWRDCASQREGCG----- -VEFNTLIPENGLMKFHQVRRVMTILFLTMVISY THGTLESVSGPKAGSRGLTSLADTFEHVIEELLDE

Bos Taurus ----------------------------- ----------------------MTILFLTMVISY THGTLESMNGPKVGSRGLTSLADTFEHVIEELLDE

Erinaceus europaeus ----------------------------- ----------------------MTILFLTMVISY THGTLESANGPKAGARDLA-LANTFEHMIEELLDE

Ailuropoda melanoleuca HTRLGICPHASVRYDCASQKKGC------ SVEFNKLILENGLIKFHQVRRVMTILFLTMVISY THGTLESVNGPKAGSRGLTSLADTFEHVIEELLDE

Mustela putorius furo ----------------------------- ---------------FHQVRRVMTILFLTMVISY THGTLESMNGPKAGSRGLTSLADTFEHVIEELLDE

Myotis lucifugus ----------------------------- ----------------------MTILFLTMVISY THGTLDSVNGPKAGSRGLTSLADTFEHVIEELLDE

**Other Placental mammals**

Dasypus novemcinctus HTCFGIYPHASV--DYTFQKKGCGMCVCA SVEFNKLIPENGLIKFHQVRRVMTILFLTMVISY THGTLESVNGPKAGSKGLPSLADTFEHVIEELLDE

Loxodonta Africana ----------------------------- --------------QFHQVRRVMTILFLTMVISY THGTLESMNGPKAGSRGLTSLADTFEHVIEELLDE

Procavia capensis ----------------------------- ---------------FHQVRRVMTILFLTMVISY THGTLESVSGPKAGSRGLTSLADTFEHVIEELLDE

Choloepus hoffmanni ----------------------------- ---------------FHQVRRVMTILFLTMVISY THGTLESMNGPKAGSKGLTSLADTFEHVIEELLDE

Echinops telfairi ----------------------------- ----------------------MTILFLTMVISY THGTLESMNGPKAGARGLPSLADTFEHVIEELLDE

Dipodomys ordii ----------------------------- -------IQGQEKRWFHQVRRVMTILFLTMVISY THGTLESVNGPQAGSRGLTSLADTFEHVIEELLDE

**Sauropsia**

Anolis carolinensis ----------------------------- ---------------------------------- TQGNLENLGGPNDATRGLTSLADTFEHVIEELLDE

Pelodiscus sinensis ----------------------------- ----------------------MTILFLTMVISY THGTLESLSGPNTGSRGLTSLADTFEHVIEELLDE

Meleagris gallopavo ----------------------------- ----------------------MTILFLTMVISY THGTLESLNGPNAGSRGLTSLADTFEHVIEELLDE

Anas platyrhynchos ----------------------SLCLVSL CLIKHSLMVLSLCAQFHQVRRVMTILFLTMVISY THGTLESLNGPNAGSRGLTSLADTFEHVIEELLDE

Ficedula albicollis ----------------------------- --------------MFHQVRRVMTILFFTMVISY PHGTLESIGGPGAGSRGLTSLADTFEHVLEELLDE

Gallus gallus ----------------------------- ----------------------MTILFLTMVISY THGTLESLTGPNAGSRGLTSLADTFEHVIEELLDE

Taeniopygia guttata ----------------------------- ----------------------MTILFFTMVISY THGTIESINGPSASSRGLTSLADTFEHVIEELLDE

Xenopus tropicalis ----------------------------- ----------------------MTILFLTMVISY THGTLESIGGLGVSGGGLPSLTDTFEHVIEELMEE

**Fish**

Poecilia Formosa ----------------------------- ----------------------MTILFLTMVISY GHGTPQSGGGPGQR-EELPSLTDTFEQVIEELLEV

Astyanax mexicanus ----------------------------- ----------------------MTILFLTMVISY GHGTPQSGGG-------LPSLTDTFEQVIEELLEA

Gadus morhua ----------------------------- ---------------FHQVRRVMTILFLTMVISY GHGTPQSGAGPGQR-GALHSLTDTFEQVLEELLEV

Takifugu rubripes ----------------------------- -------LSSVSPLQFHQVRRVMTILFLTMVISY GHGTPQSGGGPGQH-GDLPSLTDTFEQVIEELLEV

Xiphophorus maculatus -----------------------------­ ----------------------MTILFLTMVISY GHGTPQSGGGPGQR-EELPSLTDTFEQVIEELLEV

Lepisosteus oculatus ----------------------------- ---LFSPPLPPVPLQFHQVRRVMTILFLTMVISY GHGTPESGGGAPRG---LPSLADTFEQVIEELLEG

Gasterosteus aculeatus ----------------------------- -----MFQSFILPWKFHQVRRVMTILFLTMVISY GHGTPQSGGGPGQR-GELPSLTDTFEQVIEELLEV

Tetraodon nigroviridis ----------------------------- ----------------------MTILFLTMVISY GHGTPQSGGGPGQH-GALPSLTDTFEQVIEELLEV

Oreochromis niloticus ----------------------------- ----------------------MTILFLTMVISY GHGTPQSGGGPGQR-GELPSLTDTFEQVIEELLEV

Danio rerio ----------------------------- ----------------------MTILFVTMVISY GHGTPQSGGG-------LPSLTDTFEQVIEELLEV

**Others (including invertebrates)**

Macropus eugenii ----------------------------- ---------RKSEEEFHQVRRVMTILFLTMVISY THGTLESLNGPKAASRGLTSLADTFEHVIEELLDG

Monodelphis domestica ----------------------------- ----------------------MTILFLTMVISY THGTLESLNGPKAGSRGLTSLADTFEHVIEELLDE

Sarcophilus harrisii ----------------------------- ----------------------MTILFLTMVISY THGTLESLNGPKAGSRGLTSLADTFEHVIEELLDE

Ornithorhynchus anatinus ----------------------------- ---------------------------------- -----------------------------------

Latimeria chalumnae ----------------------------- --------------QFHQVRRVMTILFLTMVISY THGTLESIGGPKASSRGLTSLADTFEHLLEEILEG

Petromyzon marinus ----------------------------- ---------LSQSRQFLQGQRVMRFVLLTMLTMF SDDSARTLLIPLLREMLKEEVGWGDSHPRRARDTG

Boxes mark the identified non-synonymous variants. Yellow highlighted spots, mark amino acids which are different from human (Modified from www.ensembl.org)

**Text S1: Functional *in vitro* analyses for p.Met215Ile *MC4R***

Met215Ile *MC4R*, wild type *MC4R* and *TSHR* (positive control in ERK assay) were cloned in a pcDps expression vector. According to the manufacturer’s protocol the different vectors were transiently transfected in HEK 293 cells with Metafectene (Biotex, Munich, Germany). 48h after transfection, HEK293 cells were stimulated with an increasing concentration of α-melanocyte stimulating hormone (α-MSH; 0.1nM-1000nM) and its potent analogue, [Nle[4], D-Phe[7]]-α-MSH (NDP-α-MSH; 0.01nM-100nM) to test signal transduction properties. The cAMP assay was performed with cAMP AlphaScreen (Perkin Elmer). HEK293 cells were stimulated for 40 min at 37 °C. The reaction was stopped by adding lysis buffer for 2 h at 4°C on a shing platform. Determination of cAMP by AlphaScreen technology was done according to the manufactures` protocol. For measurement of MAP kinase activation HEK293 cells were stimulated with α-MSH or NDP-α-MSH for 6h at 37°C. After that cells were lysed for 15min at RT on the shaker and subsequently measured

Cell surface ELISA studies were performed with N-terminally HA-tagged mutant and wild type MC4R as previously described [62]. 72h after transfection, COS-7 cells were fixed in 4% formaldehyde, then incubated with biotin-labeled anti-HA monoclonal antibody (Roche, Mannheim, Germany) and after the incubation with streptavidin-labelled peroxidase (Dianova, Hamburg, Germany) color reaction was conducted in a buffer containing 0.1% H_2_O_2_ and 10 mg O-phenylendiamine. Measurements were done using an Anthos reader 2001 (Anthos Labtech Instruments, Salzburg, Austria). All data of the functional analyses were obtained from at least three independent experiments.
